# Counting coins in the dark—Austrian, German, and Swiss medical students’ perceptions of radiology

**DOI:** 10.1007/s00330-025-11395-6

**Published:** 2025-03-06

**Authors:** Magdalena Seng, Hanns-Christian Breit, Paul Hehenkamp, Christoph Johannes Zech, Drilona Lumi, Ricardo Donners

**Affiliations:** 1 Department of Radiology and Nuclear Medicine, University Hospital Basel, University of Basel, Basel, Switzerland; 2 University of Applied Sciences and Arts Northwestern Switzerland FHNW, School of Education, Institute for Special Education and Psychology, Windisch, Switzerland

**Keywords:** Surveys and questionnaires, Students (Medical), Radiology, Artificial intelligence, Curriculum

## Abstract

**Objectives:**

To investigate the overall attitude of medical students towards radiology, their perceptions of the university curriculum, the clinical relevance, and the prospects within the specialty, as well as identify reasons for and against choosing a radiologist’s career.

**Materials and methods:**

An 18-question survey was distributed among 21 universities in Germany, Switzerland, and Austria in January 2024. It was returned by 1184 medical students (753 female, 427 male, 2 diverse, 2 others) within one month. The survey encompassed sociodemographic data, questions regarding the curriculum, the perceived clinical relevance of radiology, as well as job attractiveness, students’ expectations regarding salary and work-life balance, the role of artificial intelligence (AI), and students’ outlook on the future.

**Results:**

Seven hundred and sixteen (60%) participants expressed a desire for increased exposure to radiology within the medical curriculum and 1177 (99%) acknowledged the clinical importance of radiology. However, 755 (64%) students definitively ruled out pursuing a career as a radiologist, citing limited patient interaction as the primary deterrent (*n* = 725). Notably, 85% of participants considered the potential salary and work-life balance in radiology as favorable compared to other medical disciplines. Furthermore, 396 students (33%) were discouraged by contemporary AI developments from pursuing a career in radiology.

**Conclusion:**

While medical students recognize the clinical importance of radiology, the majority does not consider becoming radiologists. The findings suggest a need for educational initiatives to address misconceptions and enhance the attractiveness of radiology as a viable career option for medical graduates.

**Key Points:**

***Question*** *The majority of medical students do not consider pursuing a career as radiologists though they recognize the clinical relevance and importance.*

***Findings*** *Students hesitate to pursue it as a career due to the perceived lack of patient interaction and concerns regarding the potential impact of AI.*

***Clinical relevance*** *Radiologists’ workload is increasing globally. However, the available workforce has remained relatively stable. Thus, recruiting future doctors into the specialty is extremely important. Therefore, insights into medical students’ contemporary perceptions of radiology are valuable in addressing reservations and misconceptions.*

## Introduction

The demand for diagnostic imaging and minimally invasive, image-guided procedures is increasing globally [1]. Many radiology departments have experienced a dramatic rise in the volume and complexity of examinations over the last decade [2], while the size of the radiology workforce has increased only minimally [3, 4]. Consequently, radiology departments are faced with the challenge of balancing a relative shortage of skilled professionals against the continually growing workload. This dissonance has profound implications for healthcare delivery, as well as the working conditions of the individual radiologist, affecting overall job satisfaction and resulting in a rising number of stress-related morbidities such as burnout [5, 6].

Several factors have led to increased utilization of medical imaging, including advances in imaging software- and hardware, allowing for faster acquisition of more complex examinations and more precise diagnoses of many medical conditions. Moreover, there is a trend of imaging being performed before or instead of clinical examinations, adding additional strain to radiology departments [7]. The emergence of artificial intelligence (AI)-based reporting algorithms may offer a solution to the described dilemma of an increased workload faced by a constant or even decreasing workforce. Accordingly, several deep learning algorithms are commercially available to support radiology practice, highlighting lung nodules, intracranial hemorrhages, and pulmonary embolisms among others [8, 9]. However, none of the AI developments thus far can replace radiologists, leaving the workforce shortage issue unresolved. Moreover, misconceptions of AI replacing radiologists may even deter future prospects from a career in radiology [10].

As such, an increase in the number of radiologists is needed to tackle the challenges lying ahead. Based on the authors’ experiences in their home institutions the recruitment process falls short of the actual need and most medical students do not consider radiology as a desired field of work. This may be due to a lack of education on contemporary radiology within the local university curricula, outdated views on radiologists’ work not actively changed during university, as well as dismal forecasts on the future due to a possible AI takeover [11]. Although more radiology trainees are needed, little research has been invested to explore why the interest in radiology as a specialty has not grown despite the rise in examinations and its crucial role in patient care. Accordingly, this study aims to understand medical students’ attitudes towards and their perception of the discipline by using a comprehensive 18-question survey distributed among universities in Germany, Switzerland, and Austria.

## Methods

The survey was distributed via an openly accessible link, and participation was entirely voluntary and anonymous. Due to the anonymous nature of the non-validated survey, an ethics committee approval was deemed not necessary. A cross-sectional survey was conducted to capture the perspectives and opinions of medical students regarding radiology as a medical specialty. The standardized questionnaire comprising 18 questions was created using the SurveyMonkey© (SurveyMonkey Europe UC) web service and a hyperlink for online access was generated to access the survey shown in Table 1. The survey was distributed in German language among medical students from 22 universities in Switzerland, Austria, and Germany. Contact was established primarily through the deans of studies or medical student associations with all medical universities in these countries, resulting in responses from two Swiss universities, one Austrian university, and 18 German universities, listed in Table 2.

**Table 1:** Questionnaire

Question	Response option
1. What year are you currently in your medical studies?	First-year
	Second year
	Third year
	Fourth-year
	Fifth year
	Sixth year and beyond
2. Which gender do you identify with?	Female
	Male
	Diverse
	None of the above
3. Where do you study medicine?	Open-ended question
4. What was your exposure to radiology so far?	None
	Lectures and seminars
	Internship, clinical traineeship
	Elective clerkship
	Doctoral candidates, graduate thesis
	Friends, relatives
5. Would you like more or less radiology in your medical curriculum?	Certainly more
	A bit more
	Neutral
	A bit less
	Certainly less
6. How would you rate the importance of radiology in current clinical practice?	Not important at all
	Slightly important
	Moderately important
	Very important
	Extremely important
7. What comes to your mind when you think of radiology and working as a radiologist?	Open-ended question
8. Can you envision yourself becoming a radiologist in the future?	Definitely yes
	Probably yes
	Unsure
	Probably no
	Definitely no
9. What factors do you believe influence medical students in choosing a career in radiology? (multiple response)	Patient contact
	Work-life balance
	Private practice
	Salary and benefits
	AI, Interest in technologies
	Opportunities for research in the field
	Radiation
	Other (please specify)
10. What factors do you believe influence medical students in avoiding a career in radiology? (multiple response)	Patient contact
	Work-life balance
	Private practice
	Salary and benefits
	AI, Interest in technologies
	Opportunities for research in the field
	Radiation
	Other (please specify)
11. How attractive do you find radiology as a career option compared to other medical specialties?	Much less attractive
	Less attractive
	Neutral
	More attractive
	Much more attractive
12. What additional measures or initiatives do you think could make radiology more appealing to medical students?	Open-ended question
13. How would you rate the salary prospects in the field of radiology?	Much better than in other specialties
	Better than in other specialties
	Comparable to other specialties
	Worse than in other specialties
	Much worse than in other specialties
14. What impact do you think will advancements in AI have on radiology?	Positive—doctors and AI will complement each other
	Negative—AI will replace radiologists in the future
	No influence
	I don’t know
	Other (please specify)
15. In considering your future career, please indicate the number of hours per week you envision yourself working.	More than 50 h per week
	41–50 h per week
	31–40 h per week
	20–30 h per week
	Less than 20 h per week
16. How do you assess the work-life balance in radiology compared to other specialties?	Much better
	Better
	Comparable
	Worse
	Much worse
17. In your view, what are the future prospects for radiologists?	Very optimistic
	Optimistic
	Neutral
	Pessimistic
	Very pessimistic
18. What prevents medical students from choosing radiology as a profession?	Open-ended question

**Table 2:** Distribution of medical students among universities

University	Number of respondents (*n*, %)
University of Basel (CH)	*n* = 212 (18%)
University of Bern (CH)	*n* = 69 (6%)
University of Graz (A)	*n* = 20 (2%)
University of Duisburg-Essen (GER)	*n* = 199 (17%)
Justus Liebig University Gießen (GER)	*n* = 159 (13%)
Johannes Gutenberg University Mainz (GER)	*n* = 117 (10%)
Charite—Universitätsmedizin Berlin (GER)	*n* = 107 (9%)
University of Münster (GER)	*n* = 102 (9%)
Albert-Ludwigs University Freiburg (GER)	*n* = 36 (3%)
Ruhr-University of Bochum (GER)	*n* = 36 (3%)
Saarland University (GER)	*n* = 33 (2%)
Heinrich-Heine University Düsseldorf (GER)	*n* = 21 (2%)
Julius-Maximilians-University Würzburg (GER)	*n* = 19 (2%)
Heidelberg University (GER)	*n* = 14 (1%)
Technical University of Munich (GER)	*n* = 14 (1%)
RWTH Aachen University (GER)	*n* = 8 (1%)
University of Ulm (GER)	*n* = 2 (0%)
Philipps-University Marburg (GER)	*n* = 2 (0%)
University of Bonn (GER)	*n* = 1 (0%)
Eberhard Karls University Tübingen (GER)	*n* = 1 (0%)
University of Greifswald (GER)	*n* = 1 (0%)
Others	*n* = 11 (1%)

In total, 1184 students (753 females, 427 males, 2 diverse, 2 others) returned the questionnaire. Given the number of enrolled students across all contacted universities as potential participants, this corresponds to an overall response rate of 1.7% across all participating institutions. Inclusion criteria required that individuals be currently enrolled in a medical faculty. Data collection took place between the 1st and 31st January 2024.

Questions pertaining to the clinical relevance of radiology, the possibility of choosing radiology as a specialty, the attractiveness of the field, the expectations regarding the salary and work-life balance, the role of AI, and prospects in radiology were answered on a 5-point Likert scale.

Additionally, three open-ended questions were posed, focusing on the future doctors’ associations with “radiology” as a specialty, asking for suggestions to enhance its attractiveness, and enquiring on factors deterring medical students from choosing to become radiologists.

**Fig. 1:**
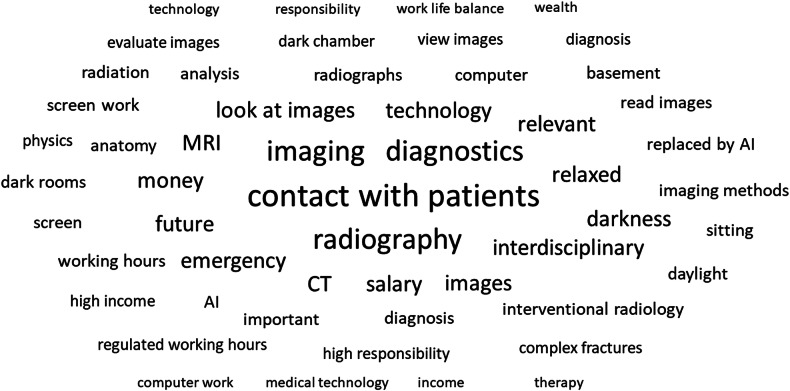
Word cloud based on the responses to question no. 7

**Fig. 2:**
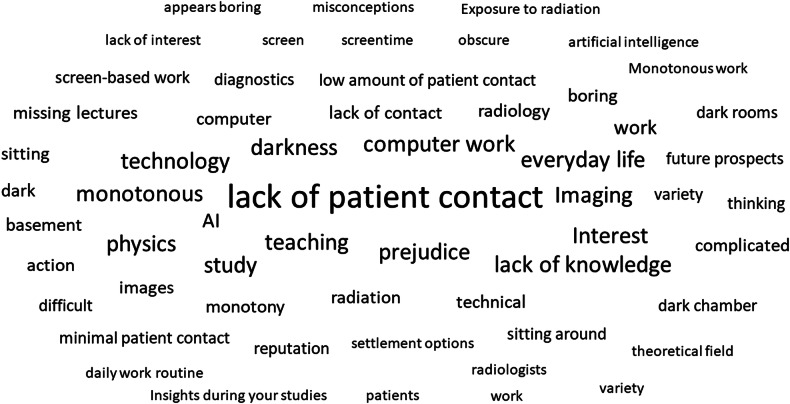
Word cloud based on the responses to question no. 18

Figure 1 (word cloud based on the responses to question no. 7) and Fig. 2 (word cloud based on the responses to question no. 18) were automatically generated using the freely available software SurveyMonkey© and translated into English.

### Statistical and subgroup analyses

The online survey tool allowed straightforward analyses, visualizing frequencies and ratios. Additional analyses and visualizations were conducted using commercially available software (Microsoft 365 Excel, version 16.85 and IBM SPSS Statistics, version 28.0.1).

Sub-analyses were performed to examine students’ interest in specializing in radiology, depending on prior radiology exposure and country.

Student answers regarding radiology’s clinical role, attractiveness, prospects, and if they could envision themselves becoming a radiologist were compared between students with little or greater previous exposure to the specialty.

Perceptions regarding the university curriculum, salary, specialty attractiveness, working hours, and impact of AI were compared between different countries of origin.

Comparisons were made using Kruskal–Wallis tests. Gender (female vs male) dependent differences in survey question responses were assessed using the Mann–Whitney *U*-test. Post-hoc Dunn–Bonferroni tests were performed to correct for multiple testing and a *p*-value < 0.05 was deemed statistically significant.

## Results

A total of 1184 medical students (753 female, 427 male, 2 diverse, 2 others) from Germany, Switzerland, and Austria returned the survey. Responders were evenly distributed across study years, but not across countries (Table 3).

**Table 3:** Sociodemographic variables: gender, year of study, and country of origin

Question	Response option	Number of responses
Gender	Female	*n* = 753
	Male	*n* = 427
Year of study	1st	*n* = 110
	2nd	*n* = 149
	3rd	*n* = 222
	4th	*n* = 245
	5th	*n* = 230
	6th and higher	*n* = 228
Country	Switzerland	*n* = 281
	Austria	*n* = 20
	Germany	*n* = 872

The majority of 755 (64%) students could not envision themselves becoming radiologists. However, 716 (60%) students expressed a desire for more radiology in their medical curriculum, while a minority of 28 (2%) students wished for less radiology teaching.

Almost all (1177, 99%) medical students considered radiology’s current clinical impact to be important, with 957 (81%) students rating it as very or extremely important and only 7 (1%) considering it rather unimportant.

### Factors for and against becoming a radiologist

**Fig. 3:**
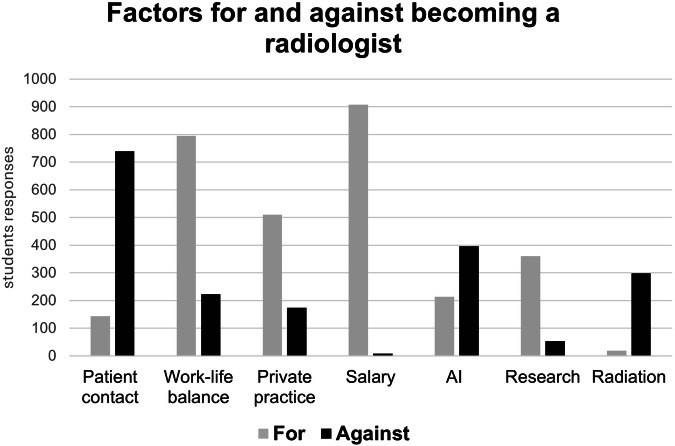
Factors for and against becoming radiologists as perceived by 1184 medical students, private practice—the ability to work in private practice as a radiologist. AI, artificial intelligence

The upsides and downsides of a career in radiology as perceived by the survey participants are visualized in Fig. 3. The main upsides for choosing a career as a radiologist named by the participants were the salary (908, 77%) and the work-life balance (795, 67%). Factors perceived to be speaking against choosing a career in radiology were the lack of patient contact (739, 62%) and the potential impact of AI (396, 33%).

### Comparison of radiology to other medical specialties as a future career

**Fig. 4:**
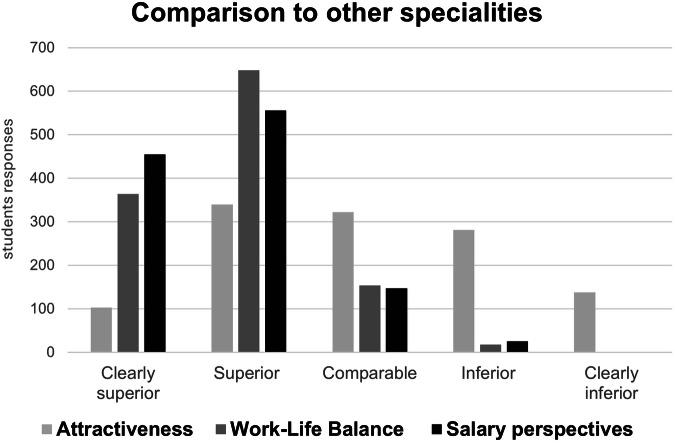
Radiology, compared to other specialties, as perceived by 1184 medical students in terms of attractiveness, work-life balance, and salary perspectives

Figure 4 summarizes the answers regarding the comparison of radiology to other medical specialties. Radiology was perceived favorably by more than three-quarters of students in terms of salary (1011, 85%) and work-life balance (1012, 85%). Regarding general work-life balance and overall working hours, a minority of 69 (6%) students expressed a desire to work more than 49 h a week, while the majority of 1063 students preferred working 30–39 h a week (548 students) or 40–49 h a week (515 students). There was no significant difference between students who could and could not envision themselves becoming radiologists regarding salary prospects in the field of radiology (*p* = 0.19). By contrast, students who could envision a career in radiology preferred to work fewer hours than their peers, who ruled out becoming radiologists (*p* < 0.05).

General specialty attractiveness was rated superior by 455 (37%) students, as comparable by 322 (27%) students, and as inferior to other specialties by 419 (35%) students.

### Future perspectives

Regarding the future of radiologists, more than half of the students (628, 53%) maintain an optimistic outlook. The majority of 714 (60%) students believed that advancements in AI yield positive impacts, resulting in a collaborative relationship between doctors and AI. However, 270 (19%) students expressed concerns about AI solutions potentially replacing radiologists in the future.

### Analysis depending on students’ prior radiology exposure

Prior radiology exposure had a significant impact on students’ perceptions of radiology’s clinical role, attractiveness, and prospects, as well as their interest in becoming a radiologist (each *p* ≤ 0.002). Students who underwent dedicated clerkships or were engaged in radiology theses reported significantly higher values regarding attractivity, prospects, clinical importance, and overall interest in becoming a radiologist (Table 4).

**Table 4:** Impact of students’ prior radiology exposure on survey responses

	Group 1% (*n*)	Group 2% (*n*)	Group 3% (*n*)	Group 4% (*n*)	*p*-values (*p*)
Students’ perception of the Importance of clinical practice					< 0.001
Extreme	19 (29)	25 (176)	34 (97)	61 (14)	
Very	56 (86)	56 (404)	50 (142)	39 (9)	
Moderate	24 (37)	19 (138)	16 (45)	0 (0)	
Slightly	2 (3)	0 (2)	1 (2)	0 (0)	
Not	0 (0)	0 (0)	0 (0)	0 (0)	
Students perception of radiology attractivity compared to other specialties					< 0.001
Much more	8 (13)	6 (42)	14 (40)	35 (8)	
More	24 (37)	27 (195)	33 (95)	57 (13)	
Neutral	30 (46)	29 (205)	25 (70)	4 (1)	
Less	25 (38)	26 (188)	19 (54)	4 (1)	
Much less	14 (21)	13 (90)	9 (27)	0 (0)	
Students’ views of the prospects of radiologists					= 0.002
Very optimistic	5 (8)	7 (52)	10 (27)	17 (4)	
Optimistic	38 (59)	44 (319)	43 (123)	65 (15)	
Neutral	40 (62)	34 (245)	37 (105)	13 (3)	
Pessimistic	16 (25)	13 (93)	9 (27)	4 (1)	
Very pessimistic	1 (1)	1 (10)	1 (4)	0 (0)	
Can students envision themselves becoming radiologists					< 0.001
Definitely yes	5 (7)	2 (16)	13 (37)	52 (12)	
Probably yes	25 (38)	18 (127)	22 (62)	26 (6)	
Unsure	11 (17)	10 (72)	12 (33)	8 (2)	
Probably no	42 (65)	45 (325)	35 (100)	8 (2)	
Definitely no	18 (28)	25 (180)	19 (54)	4 (1)	

Based on question 4, responses were summarized in groups. Group 1 = no exposure; Group 2 = lectures, seminars, friends, relatives; Group 3 = internship, clinical traineeship; Group 4 = doctoral candidates, graduate thesis

*n* number of respondents, % percentage, *p* *p*-values

### Analysis depending on students’ country of studies

German students wished significantly more for increased radiology in the curriculum and were more commonly considered to become radiologists (each *p* ≤ 0.001) when compared to Austrian and Swiss medical students (Table S1).

### Analysis depending on students’ gender

The survey revealed significant gender differences regarding the survey answers (Table 5). Females reported lower salary expectations compared to other specialties (*p* < 0.001) and preferred to work fewer hours (*p* < 0.001). Males rated the work-life balance in radiology more favorably (*p* = 0.008). Females were more optimistic about the prospects in radiology (*p* = 0.022).

**Table 5:** Analysis depending on students’ gender

	Gender (f/m)	Number (*n*)	Most common responses	*p*-values (*p*)
13. How would you rate the salary prospects in the field of radiology?	f	753	Better than in other specialties	< 0.001
	m	427	Much better than in other specialties	
15. In considering your future career, please indicate the number of hours per week you envision yourself working.	f	753	31–40 h per week	< 0.001
	m	427	41–50 h per week	
16. How do you assess the work-life balance in radiology compared to other specialties?	f	753	Better	= 0.008
	m	427	Better	
17. In your view, what are the future prospects for radiologists?	f	753	Optimistic	= 0.022
	m	427	Optimistic	

Based on questions 13, 15, 16, and 17 responses were divided into genders female (f) and male (m)

*n* number of respondents, *p* *p*-values

### Associations and students’ suggestions for improvement

By responding to the open-ended questions, the students’ associations with radiology revealed a wide array of perspectives and perceptions, many mirroring the above-described answers to the multiple-choice questions. A majority of responders emphasized the concerns regarding limited patient interaction (*n* = 725), thus reducing the specialty’s appeal as a future career option. Moreover, students reflected on unappealing working conditions (*n* = 150) (“sitting alone in darkness all day only interpreting images”).

A large proportion of medical students expressed concerns about the impact of AI on daily radiology practices in the open-ended questions. They mostly feared that AI might threaten radiologists’ future job prospects: “In the medium term, I foresee human-performed diagnostic radiology being at a disadvantage compared to artificial intelligence. With AI advancing quickly and having access to vast databases, it will soon be better than humans.” Some indicated that AI could lead to radiological tasks being taken over by other specialties, resulting in an overall reduced need for radiologists.

Several participants expressed a lack of sufficient knowledge about the practical aspects of this career and wished for “increased student exposure and improved teaching involving direct patient interaction”.

Another student wished for “Bedside radiology with patient contact. Generally, more varied insights than the typical imagination of ‘sitting in a dark chamber’.” The student went on to describe “On the other hand, radiology is a specific discipline (like pathology) and, apart from interventional radiology and radiotherapy, it’s heavily focused on diagnostics. Students primarily dedicated to patient care and treatment will never be convinced. That’s inherent to the discipline”.

## Discussion

This 18-question survey, distributed among 21 European universities (Germany, Switzerland, Austria) revealed that the majority of the participating 1184 medical students do not envision themselves becoming radiologists. This contrasts with the increasing demand for radiology professionals due to the globally rising number and complexity of imaging examinations [12]. In our study, the primary factors listed against choosing a career in radiology were the perceived lack of patient interaction, missing representative insight during university training, and the impression that AI developments may render the radiologist obsolete. Salary and work-life balance were highlighted as positive aspects, making the field more appealing in comparison to other specialties in these regards. Most students were aware of the important clinical impact of radiology, but pointed out that it only plays a minor role in the university curriculum and wished for more dedicated hands-on teaching.

Although most of our study participants could not picture themselves becoming radiologists, there appears to be no general rejection or disinterest in the specialty. Sixty percent of the participants in our study group indicated a desire to incorporate more radiology into their curriculum. Accordingly, a UK study found that radiology is disproportionately underrepresented in total medical curriculum hours [13]. A Canadian study demonstrated similar findings, recommending early exposure of medical students to radiology in their curriculum to generate interest [14, 15]. This suggestion aligns with other authors, who highlight the need for enhanced radiology education for undergraduates [16, 17]. These reports and the findings from the presented study mirror the authors’ own experiences as students, as well as lecturers, that radiology is underrepresented in the contemporary medical curriculum.

Although a survey conducted in the UK from 2010 to 2020 showed that increased exposure to radiology teaching does not influence students’ decision to pursue a career in radiology [18], we assert that it needs to play a greater role in the medical curriculum given the growing prevalence of imaging and its role in patient management. This disparity should be addressed by medical faculties. The presented study highlights, that students with greater exposure to the specialty, are more likely to pursue a career as radiologists.

Perhaps unsurprisingly and enhanced by the relatively little exposure of medical students to radiology during their studies, the perception of radiology is still largely based on stereotypes, thereby diminishing the appeal of the field [19]. While medical students expect a high salary and good work-life balance, concerns persist about limited patient interaction and a perceived absence of concern for patient outcomes [20].

In our study, the scarcity of patient contact was the most stated negative aspect of radiology, both in closed and open-ended questions. Previous authors described that US radiology residents chose their careers primarily based on universities’ hands-on courses [21]. Consequently, we believe increasing opportunities for patient interaction in radiology courses and more interactive hands-on experiences such as active participation in sonographic examinations, independent reporting, or using interventional training simulators may lead more future doctors towards a career in radiology. Additionally, one frequently cited concern is the impact of AI [22]. The survey revealed through open-ended questions, that some medical students maintain the opinion that AI advancements may render diagnostic radiologists obsolete in the near future. Previous studies have shown that some clinicians share these opinions [23–25]. Even though AI has made major advances in radiology, radiologists’ work is highly complex and not easily replicated by machine learning [26, 27]. The infamous quote by the Turing award winner Geoffrey Hinton: “We should stop training radiologists now. It’s completely obvious that within five years, deep learning is going to do better than radiologists” in 2016 highlights the misconceptions concerning AI in radiology among society and even highly educated researchers. As such, it is easy to imagine how medical students may be influenced and stay away from a career in radiology. Once more it becomes clear that it is important to invest in education and provide awareness about current technological advancements and benefits.

This study has several limitations. Firstly, the curriculum varies between universities and countries, however, we found few statistically significant differences between students’ responses when compared between countries. Secondly, the survey was only distributed in German-speaking regions, limiting its generalizability to other universities and regions. Thirdly, the influence of individual universities and their teaching methods may be overrepresented, necessitating further investigation. Additionally, open-ended questions may vary in terms of time and patience required, potentially leading to biased responses.

In conclusion, medical students are aware of the clinical relevance of radiology in modern medicine, but are reluctant to pursue a radiologist’s career. Lack of patient interaction, limited hands-on experiences in the curriculum, and a potential AI takeover contribute to its limited attractiveness. More radiology courses in the curriculum and dedicated hands-on training should be pursued to increase interest in our specialty.

## Supplementary information

ELECTRONIC SUPPLEMENTARY MATERIAL

## Abbreviations

AI: Artificial intelligence
*n*: Number of respondents
*p*: *p*-values
