# Supplementary material for: Counting coins in the dark—Austrian, German, and Swiss medical students’ perceptions of radiology
Source: Eur Radiol. 2025 Mar 6;35(9):5440–9. doi: 10.1007/s00330-025-11395-6 (PMC12350572; doi:10.1007/s00330-025-11395-6)
Supplement: Supplementary file 1 — ELECTRONIC SUPPLEMENTARY MATERIAL [file 330_2025_11395_MOESM1_ESM.pdf]

Counting coins in the Dark – Austrian, German and Swiss medical students’ perceptions of Radiology
ELECTRONIC SUPPLEMENTARY MATERIAL

	**Germany % (n)**	**Switzerland % (n)**	**Austria % (n)**	**p-values (p)**
Would you like more or less radiology in your medical curriculum?				< 0.001
certainly more	21 (184)	12 (33)	29 (6)	
a bit more	43 (376)	39 (109)	38 (8)	
neutral	35 (312)	44 (122)	29 (6)	
a bit less	1 (12)	4 (11)	5 (1)	
certainly less	0 (1)	1 (3)	0 (0)	
Can you envision yourself becoming a radiologist in the future?				< 0.001
definitely yes	8 (70)	1 (2)	0 (0)	
porobably yes	22 (191)	14 (39)	14 (3)	
unsure	11 (99)	9 (24)	5 (1)	
probably no	39 (347)	49 (135)	48 (10)	
definitely no	20 (178)	28 (78)	33 (7)	

Based on question 6 and 8, responses were divided into countries (Germany, Switzerland, Austria).
n = number of respondents; % = percentage, p = p-values
